# Long non‐coding RNA MALAT1 regulates retinal neurodegeneration through CREB signaling

**DOI:** 10.15252/emmm.202216660

**Published:** 2022-12-07

**Authors:** Jin Yao, Xiao‐Qun Wang, Yu‐Jie Li, Kun Shan, Hong Yang, Yang‐Ning‐Zhi Wang, Mu‐Di Yao, Chang Liu, Xiu‐Miao Li, Yi Shen, Jing‐Yu Liu, Hong Cheng, Jun Yuan, Yang‐Yang Zhang, Qin Jiang, Biao Yan

**Partial Retraction of:** *EMBO Mol Med* (2016) 8: 346–362. DOI: 10.15252/emmm.201505725| Published online 10 March 2016

## Journal statement

This partial retraction replaces the editorial note from February 2022. Journal editors became aware of potential image aberrations in the figures in August 2021. At the time, the editors had notified the authors' institution to request an investigation into the aberrations. The investigation concluded that image processing errors affecting Figs 3B and 7C and D, Appendix Figs S2 and S5 resulted in images being mislabeled and misrepresented. Readers were alerted in February 2022 that the authors were repeating experiments to address aberrations in the figures. The committee determined that the conclusions derived from the repeat experiments were consistent with the conclusions presented in the original paper. The data and analysis from the repeated experimentation were peer reviewed by the journal in April 2022 by a retinal degeneration expert and statistics expert, and these referees determined that the conclusions reported in the original article were consistent with the repeated experimental data. The source data for these experiments are published with this notice.

**Figure 3:**
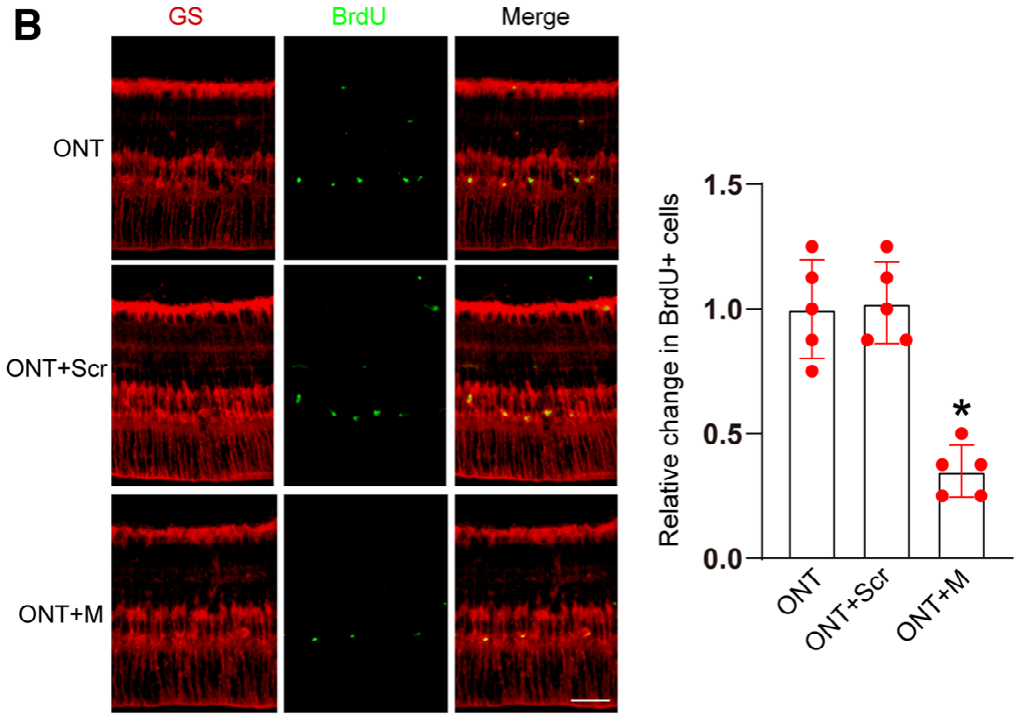
MALAT1 knockdown reduces the proliferating ability of Müller glia BFour‐month‐old male C57BL/6J mice received an intravitreous injection of scrambled (Scr) shRNA or MALAT1 (M) shRNA, or left untreated for 1 week. Then, ONT models were built, and BrdU (50 mg/kg) was injected at day 7 after building ONT model. At day 14 after building ONT model, these mice were killed and then stained with BrdU and glutamine synthetase (GS) to detect the proliferation ability of Müller glia. Data are presented as mean ± standard deviation (SD), *n* = 5 animals per group; scale bar, 100 μm; one‐way ANOVA followed by Dunnet's multiple comparison test; *P = 0.0002 (ONT + M vs. ONT); *P* = 0.8327 (ONT + Scr vs. ONT); *P = 0.0013 (ONT + M vs. ONT + Scr). The representative images of one replicate experiment and statistical results were shown. Source data are available online for this figure.

**Figure 7:**
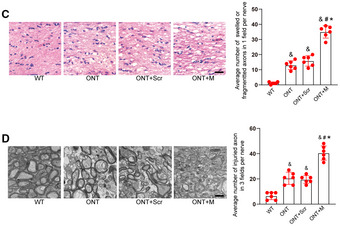
MALAT1 knockdown aggravates the injury of optic nerves CDegeneration of axons in the injured optic nerves was detected by hematoxylin and eosin (HE) staining. The photographs were taken at 40× magnification for each nerve (central portion of optic nerve). Data are presented as mean ± standard deviation (SD), *n* = 6 animals per group; scale bar, 20 μm; one‐way ANOVA followed by Dunnet's multiple comparison test; ^&^ *P* = 2.14 e‐6 (ONT vs. WT); ^&^ *P* = 1.29 e‐6 (ONT + Scr vs. WT); ^&^ *P* = 2.02 e‐9 (ONT + M vs. WT); ^#^ *P* = 4.83 e‐6 (ONT + M vs. ONT); *P* = 0.1553 (ONT + Scr vs. ONT); *P = 0.00023 (ONT + M vs. ONT + Scr).DDegeneration of axons in the injured optic nerves was detected by electron microscopy. Three ultra‐thin cross sections per nerve were observed and added together to count the number of injured axons. Counting of injured axons was performed by three different investigators who were blinded to group identity and injury status. An average counting number of the three investigators was used for statistical analysis. Data are presented as mean ± SD, *n* = 6 animals per group; scale bar, 0.5 μm; one‐way ANOVA followed by Dunnet's multiple comparison test; ^&^ *P* = 0.00011 (ONT vs. WT); ^&^ *P* = 2.36 e‐5 (ONT + Scr vs. WT); ^&^ *P* = 1.24 e‐7 (ONT + M vs. WT); ^#^ *P* = 1.18 e‐5 (ONT + M vs. ONT); *P* = 0.6788 (ONT + Scr vs. ONT); *P = 0.00015 (ONT + M vs. ONT + Scr). The representative images of one replicate experiment and statistical results were shown. Source data are available online for this figure.

**Appendix Figure S2:**
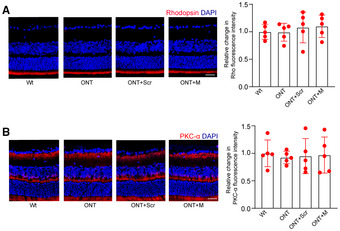
MALAT1 knockdown has no effect on photoreceptors and bipolar cells in ONT retinas A, BFour‐month‐old male C57BL/6J mice received an intravitreous injection of scrambled (Scr) shRNA or MALAT1 (M) shRNA, or left untreated for 1 week. Then, ONT models were built. Two weeks after building ONT model, retinal slices were stained with rhodopsin (Rho, A) or PKCα (B) to label photoreceptors and bipolar cells. Data are presented as mean ± standard deviation (SD), *n* = 5 animals per group; scale bar, 100 μm; one‐way ANOVA followed by Dunnet's multiple comparison test. For rhodopsin: *P* = 0.934 (ONT vs. WT), *P* = 0.584 (ONT + Scr vs. WT), *P* = 0.387 (ONT + M vs. WT), *P* = 0.562 (ONT + Scr vs. ONT), *P* = 0.380 (ONT + M vs. ONT), and *P* = 0.897 (ONT + M vs. ONT + Scr). For PKCα: *P* = 0.545 (ONT vs. WT), *P* = 0.785 (ONT + Scr vs. WT), *P* = 0.868 (ONT + M vs. WT), *P* = 0.893 (ONT + Scr vs. ONT), *P* = 0.810 (ONT + M vs. ONT), and *P* = 0.778 (ONT + M vs. ONT + Scr). The representative images of one replicate experiment and statistical results were shown.

**Appendix Figure S5:**
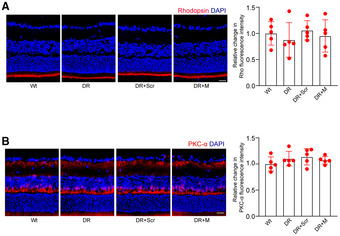
MALAT1 knockdown has no effect on photoreceptors and bipolar cells in diabetic retinas A, BThree‐month‐old male Sprague–Dawley (SD) rats received an intravitreous injection of scrambled (Scr) shRNA or MALAT1 (M) shRNA, or left untreated for 1 week. Then, the diabetic models were built. Six months after diabetes induction, retinal slices were stained with rhodopsin (Rho, A) or PKCα (B) to label photoreceptors and bipolar cells. Data are presented as mean ± standard deviation (SD), n = 5 animals per group; scale bar, 100 μm; one‐way ANOVA followed by Dunnet's multiple comparison test. For rhodopsin: *P* = 0.506 (DR vs. Wt), *P* = 0.663 (DR + Scr vs. Wt), *P* = 0.783 (DR + M vs. Wt), *P* = 0.312 (DR + Scr vs. DR), *P* = 0.718 (DR + M vs. DR), and *P* = 0.524 (DR + M vs. DR + Scr). For PKCα: *P* = 0.271 (DR vs. Wt), *P* = 0.178 (DR + Scr vs. Wt), *P* = 0.330 (DR + M vs. Wt), *P* = 0.641 (DR + Scr vs. DR), *P* = 0.669 (DR + M vs. DR), and *P* = 0.498 (DR + M vs. DR + Scr). The representative images of one replicate experiment and statistical results were shown.

Consequently, Figs 3B and 7C and D, and Appendix Figs S2 and S5 are being retracted, and the data from the repeated experiments are published with this notice.

## Author statement

We sincerely regret the image processing errors in Figs 3B and 7C and D, and Appendix Figs S2 and S5 and agree with the decision to retract these panels. All authors affirm the integrity and authenticity of the repeat data. We would like to state that the overall conclusions of this study are not affected by the partial retraction.

All authors agree with this partial retraction and apologize for their oversight and any confusion it may have caused.

## Supporting information



Source Data for Appendix

Source Data for Figure 3B

Source Data for Figure 7C

Source Data for Figure 7D

