# Supplementary material for: Long non‐coding RNA MALAT1 regulates retinal neurodegeneration through CREB signaling
Source: EMBO Mol Med. 2022 Dec 7;14(12):e16660. doi: 10.15252/emmm.202216660 (PMC9727926; doi:10.15252/emmm.202216660)
Supplement: Supplementary file 5 [file EMMM-14-e16660-s002.pdf]

EMBO
Molecular Medicine

Long non-coding RNA MALAT1 regulates retinal neurodegeneration through CREB signaling

Jin Yao, Xiao-Qun Wang, Yu-Jie Li, Kun Shan, Hong Yang, Yang-Ning-Zhi Wang, Mu-Di Yao, Chang Liu, Xiu-Miao Li, Yi Shen, Jing-Yu Liu, Hong Cheng, Jun Yuan, Yang-Yang Zhang, Qin Jiang, Biao Yan

ORIGINAL PUBLICATION DATE: 10.03.2016, EMBO Mol Med (2016) 8: 346-362
https://doi.org/10.15252/emmm.201505725

Correction timeline:

Journal contacted Authors:	9th March 2021
Authors' response to Journal:	5th September 2021
Journals' response to Authors	7th September 2021
Journal to institute	30th September 2021
Institute to journal	11th October 2021
Journal to authors	2nd November 2021
Authors to journal	4th November 2021
Authors to journal	7th February 2022
Editor to referee	11th April 2022
Referee to Journal	26th April 2022
Editor to statistics expert	29th April 2022
Statistics expert to Journal	3rd May 2022
Journal's response to authors	7th June 2022
Author's response	20th June 2022
Journal's response	15th July 2022
Author's Response	17th July 2022

Staff: Karin Dumstrei, Bernd Pulverer, Jingyi Hou, Erica Wilfong Boxheimer, Christopher Rickerby.

Report:

Authors' response to Journal. 5th September 2021

Dear editor:

We just received the following e-mail about our image aberrations in EMM-2015-05725-V4. You mentioned the following " We have carefully checked your figures and the source data you provided at publication against one another and have come across several inconsistencies that need to be addressed". Could you tell us which images have aberrations? This paper has experienced more than 4 times revision. At the beginning, the number of biolgoical repicates was less than 5. During the revision, the reviewers and editor asked us to proivde more biological repicates. We conducted more experiments to provide more replicates. This revision may lead to some inconsistencies. We are very for these errors. If required, we can provide the original data and the relevant repicates. As for the inconsistencies, we can re-conduct the relevant experiment and provide new results.

Finally, we can assure you that we have carefully conduct this experiment and do not have academic misconducts. We are so sorry for the relevant unintentional errors. We hope you can give a chance to correct these errors.

Notebly, the key findings of this paper has been verfied in the following studies published in recent years, such as

Targeting long non-coding RNA MALAT1 alleviates retinal neurodegeneration in diabetic mice （Int J Ophthalmol. 2020; 13(2): 213–219. Similar animal diease model ）

LncRNA MALAT1 facilitates inflammasome activation via epigenetic suppression of Nrf2 in Parkinson's disease （Molecular Brain volume 13, Article number: 130 (2020) also reveal the role of MALAT1 in neurodegenerative diease

Down-regulation of Long Noncoding RNA MALAT1 Protects Hippocampal Neurons Against Excessive Autophagy and Apoptosis via the PI3K/Akt Signaling Pathway in Rats with Epilepsy. Journal of Molecular Neuroscience volume 65, pages234–245 (2018 also reveal the role of MALAT1 in neurodegenerative diease)

Long Non-Coding RNA-MALAT1 Mediates Retinal Ganglion Cell Apoptosis Through the PI3K/Akt Signaling Pathway in Rats with Glaucoma. Cell Physiol Biochem 2017;43:2117–2132 (They used the same animal diease model)

Long Noncoding RNA MALAT1 and Regulation of the Antioxidant Defense System in Diabetic Retinopathy. Diabetes 2021 Jan; 70(1): 227-239. (They also used a simialr animal model

Preservation of vision after CaMKII-mediated protection of retinal ganglion cells . (Cell 2021. The authors also reported the role of CREB in retinophathy)

Best wishes

Biao Yan and Qin Jiang

Journals' response to authors 7th September 2021

Dear Dr Biao Yan and Dr. Qin Jiang,

Thank you for your message. First, I want to assure you that we are not accusing you of scientific misconduct. That said, we do note duplications within the figures and between

figures. The affected figures are 3B, 7C, 7D, S2 and S5. Please review these figures yourself and send us the related source data. Our goal is to ensure that the scientific record is accurate, and we are interested in working with you and your institute to do so.

In the mean time, would you please send us the contact information for the appropriate person at your institute?

Sincerely,

Erica Boxheimer

Journal to Institute **30th September 2021**

I am contacting you as the head of the academic committee of Eye Hospital, Nanjing Medical University, on a manuscript from Dr. Biao Yan, which was published by EMBO Molecular Medicine in 2016 (DOI 10.15252/emmm.201505725). We have recently become aware of potential image aberrations in their paper, and we conducted a standard image analysis in which we found additional inconsistencies in the figures. The detected issues concern staining image reuse within and between figures. The authors sent us source data, but we have no way to validate it.

In cases of serious image aberrations that could undermine the conclusions of the paper, we notify the authors' institution to provide an opportunity for quality control and investigation at the institutional level. We have a policy to cooperate with institutions whenever possible, and in our experience, this can help identify the causes of the apparent aberrations since institutions can directly view lab books and interview the authors.

I have attached here a summary of our image analysis results and the source data provided by the authors with my comments. I would be happy to discuss these issues in a further conversation, either via e-mail, video call or telephone. I kindly ask that you update us within the next two weeks to inform us about further steps from your side. I'm looking forward to hearing from you.

Please note that we have informed Dr. Yan in parallel that we have contacted you on this matter. We welcome your response at your earliest convenience.

Institute to Journal **11th October 2021**

Thanks for giving us an opportunity for quality control and investigation at the institutional level. As the head of the academic committee of Eye Hospital, I and my colleagues have viewed the lab books and interviewed the first and corresponding authors. We also carefully view all source data. The survey reports are shown below:

(1) For PKC and Rhodopsin staining in Fig S2 and S5.

Due to not paying enough attention for supplementary materials, they submitted the wrong assembled Fig S2 and S5, which led to image duplications among different experimental groups. About this problem, they have published the Erratum EMBO Mol Med. 2016 Apr 1;8(4):346-62. doi: 10.15252/emmm.201505725. They have provided all relevant source data, which suggested that ONT treatment or DR treatment did not affect the expression PKC and Rhodopsin. Given this conclusion is verified by two different experiments (IF staining and WB) and two different diseases models. WB experiments provide additional

evidence to prove the results of IF staining as shown in Fig S2 and S5. Thus, this error did not affect the final conclusion.

(2) For background process problems: WB background (Fig S3 GFAP, Fig S8 Tubulin) and Fig 5G untreated group for panel empty.

They have provide us the 5 different replicates of dots for Fig S3 GFAP and Fig S8 Tubulin. These replicates have similar expression pattern. These 5 different replicates were conducted at different time points and at different instruments. Thankfully, they only adjusted the background of dots, but did not process the dot bands. The final results were expressed as the relative change of band gray value compared with control group. This statistical method was not affected by the background of WB. Thus, the final conclusion was not affected.

For Fig 5G untreated group, this problem is associated with the processing of IF images from gray model to RGB model. The authors also provided the relevant source data. Generally, there was no apoptotic cells in untreated group. Thus, this inappropriate processing would not affect the final conclusion.

(3) For Fig 7C and D, Fig 3B and S7B (GS and BrdU double staining)

The authors have provided the source data for these images. There were a lot of original images for these experiments. During the selection of representative images, they mistakenly selected the representative images from the same group, which led to the wrong choices of representative images. In addition, they took more than one photos for a slice. They did not move the slice enough between the intervals of photo capture, which led to the duplications between different photos.

For 7C and D, the authors have provided the enough replicates and the relevant source data. For Fig 3B and S7B, they have also provided other replicates. In addition, the results of nestin and vimentin in Figure 3C, 3D, and S8 could provide additional evidence to support their findings in Fig 3B and S7B. Given the final conclusion was determined by the bar graphs but not the representative images, the wrong selected representative images did not affect the final conclusion.

The third-party verification: To further investigate whether the conclusion is believable, the authors have provided the third-party evidences to verify the main findings in this paper.

Finding 1: MALAT1 knockdown reduces reactive gliosis, Müller cell activation, and RGC survival in vivo and in vitro.

Which was supported by the following papers:

1) Long Non-Coding RNA-MALAT1 Mediates Retinal Ganglion Cell Apoptosis Through the PI3K/Akt Signaling Pathway in Rats with Glaucoma. Cell Physiol Biochem 2017;43:2117–2132 (They used the same animal disease model, ONT model)

2) Long Noncoding RNA MALAT1 and Regulation of the Antioxidant Defense System in Diabetic Retinopathy. Diabetes 2021 Jan; 70(1): 227-239. (They also used a similar animal model, DR model)

3) Targeting long non-coding RNA MALAT1 alleviates retinal neurodegeneration in diabetic mice （Int J Ophthalmol. 2020; 13(2): 213-219. Similar animal disease model, DR model）

Finding 2: CREB signaling is involved in retinal neurodegeneration

Which was supported by the following papers:

1) Preservation of vision after CaMKII-mediated protection of retinal ganglion cells (Cell 2021 The authors also reported the role of CREB in retinopathy)

2) PACAP attenuates optic nerve crush-induced retinal ganglion cell apoptosis via activation of the CREB-Bcl-2 pathway (Journal of Molecular Neuroscience, 2019 They also reported that CREB in retinal ganglion cell apoptosis)

Taken together, the authors could provide the source data for these relevant images and there were no data integrity issue. However, due to their careless and paying insufficient attention to supplementary materials, they have made the relevant errors.

Subsequently, we suggest that the journal can allow the authors to publish an erratum to correct these errors. If required, they could use the stored samples to re-conduct the relevant experiments to verify the conclusion. I and our colleagues could supervise the process of re-conducted experiments.

Journal to authors **2nd November 2021**

Thank you for your patience. We have now had the chance to re-discuss your paper and would like to request you to repeat the experiments under the supervision of the investigative committee, as suggested by Dr. Shang. After the experiments are done we can decide how to proceed based on the results. How long would it take you to repeat the experiments with the original samples?

Authors to Journal **4th November 2021**

Thank you for giving us the chance to repeat the experiments under the supervision by Dr. Shang.

Today, we had a meeting and discussed with the graduate students who will be responsible for the subsequent experiments. They tell us that they will take about 3 months to complete all experiments. The time required for the relevant experiments as shown below:

- Western blots: 2-3 weeks for the relevant blots with five replicates;
- PI staining: 2-3 weeks including cell thawing and PI staining with 4 replicates;
- IF staining and HE staining for tissue slices: about 3 weeks;
- Electron microscope experiments: about 4-5 weeks. We required advance reservation for electron microscope in another institute (about 4-week advance reservation).

Today, they also carefully checked the original samples. Fortunately, we have all relevant protein samples and embedded tissue samples. We are afraid of some antibodies may not work because these antibodies were purchased in 2014. If they could not work, we will buy the same antibody from the same company with the same catalogues number. Due to the antibody is usually not in stock in China and require imports, it may take more time due to the effects of Covid-19.

Taken together, we will try our best to complete all experiments within three months. If more questions, please feel free to contact us.

Authors to Journal **7th February 2022**

Thank you for giving us the chance to repeat the experiments under the supervision by Dr. Shang. It took about 3 months and we have repeated all relevant experiments. All experiments were repeated and analyzed by two different students. The results can be recogizned as the Third party certification. Moreover, all results can repeat the published data and are consistent with the previous conclusions.

For Fig 3B, GS and BrdU staining

Problem:

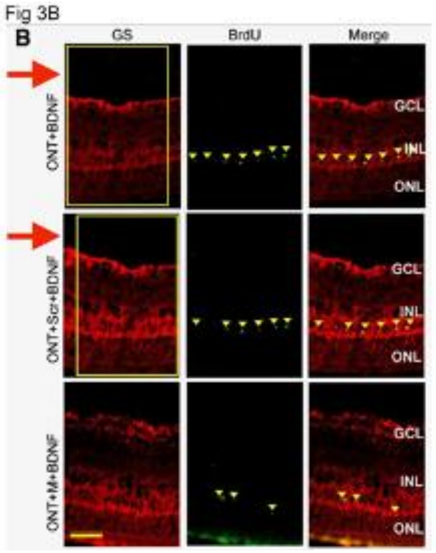

We repeated the relevant experiment to determine whether MALAT1 knockdown affected the regenerative ability of Müller cells. This experiments included 3 groups: ONT (traumatic injury), ONT+Scramble shRNA (Scr), and ONT+MALAT1 shRNA (M). We labeled the proliferating cells using BrdU staining. Intravitreous injection of MALAT1 shRNA significantly reduced the number of BrdU-labeled cells in the ONT retinas. Moreover, BrdU-labeled staining cells were overlapped with glutamine synthetase (GS) staining, suggesting that **MALAT1 knockdown affects Müller cell proliferation** as shown below. **The result can repeat the published data and is consistent with the previous conclusion.**

Experimental results:

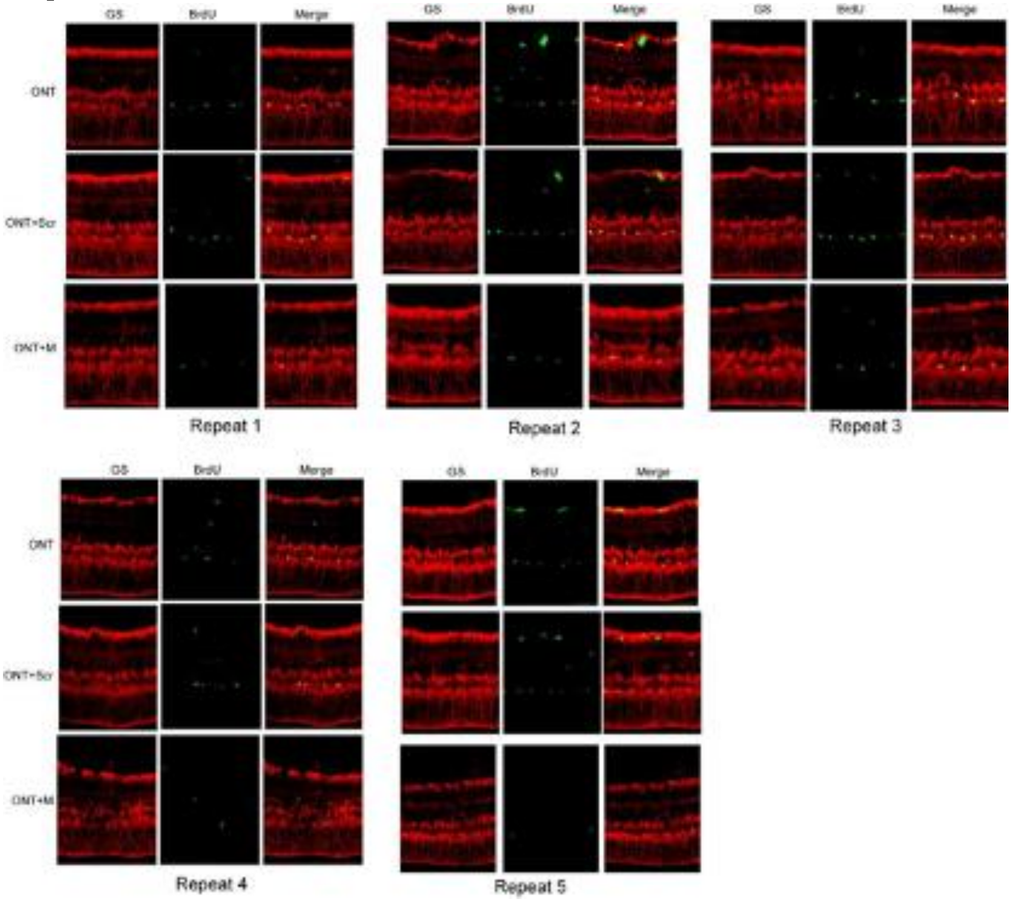

For 7C and 7D, HE staining and electron microscopy

Problem:

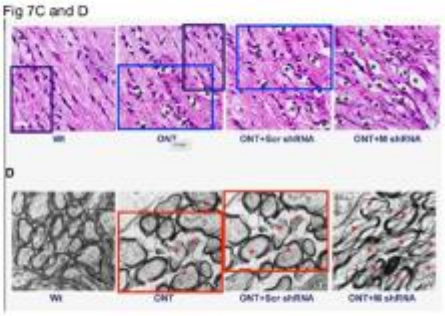

Response:

In the experiment, we repeated the relevant experiments to detect the degeneration of axons in the injured optic nerves by HE staining (C) and electron microscopy (D) in

4 different groups, including untreated group (Wt), ONT (traumatic injury), ONT+Scramble shRNA (Scr), and ONT+MALAT1 shRNA (M).

Hematoxylin-eosin (HE) staining revealed that the axons of ONT retinas have more swellings and/or fragmentation. MALAT1 knockdown further aggravated the damage of RGC axons. **The result can repeat the published data and is consistent with the previous conclusion.**

Experimental results:

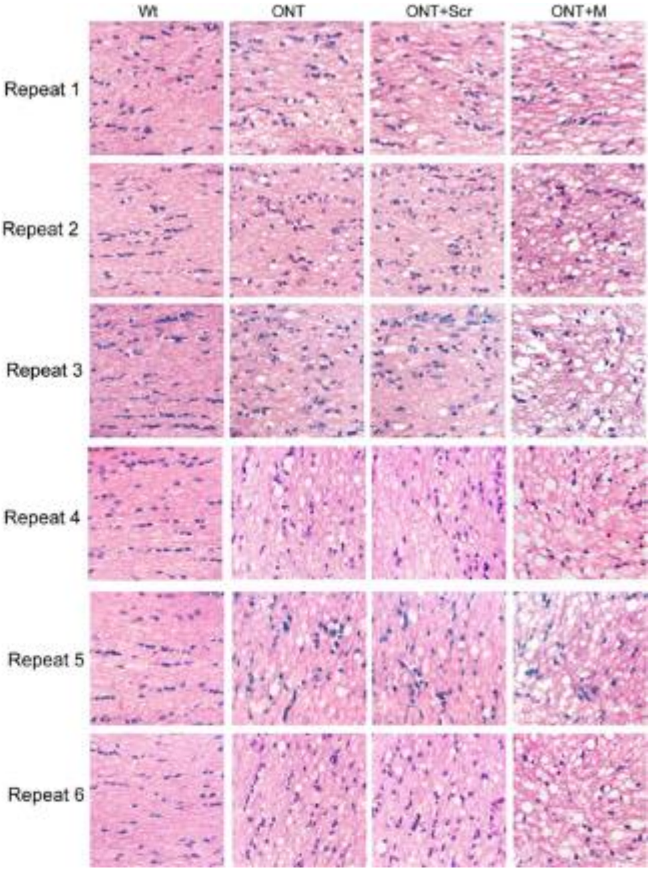

Experimental results:

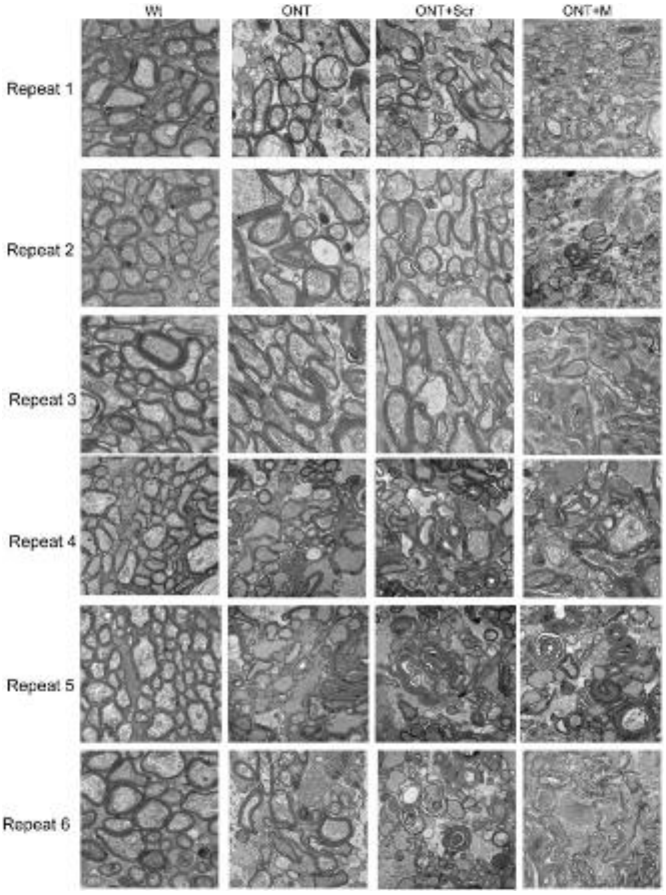

For 5G: Detection of PI-positive RGCs by PI staining

Problem:

Fig 5G "untreated" panel empty

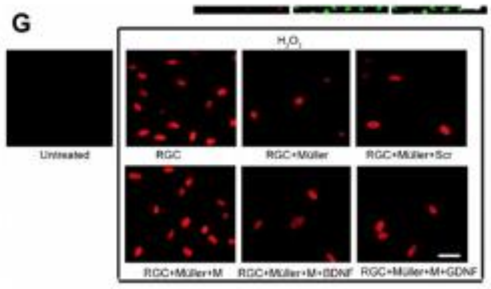

Response:

Primary RGCs were co-cultured with Müller cells. Müller cells were transfected with MALAT1 (M) siRNA or scrambled (Scr) siRNA, and then treated with or without BDNF or GDNF. After these treatments, the experimental groups were exposed to H_2O_2 (50 lm) for 48 h. PI staining was performed to detect the dead or dying RGCs. The result showed that <u>Müller cell co-culture significantly decreased the number of</u>

apoptotic RGCs, while MALAT1 knockdown in Müller cells obviously attenuated this protective effect. Exogenous BDNF or GDNF administration could eliminate the adverse effect of MALAT1 knockdown. **The result can repeat the published data and is consistent with the previous conclusion.**

Experimental results:

Repeat 1

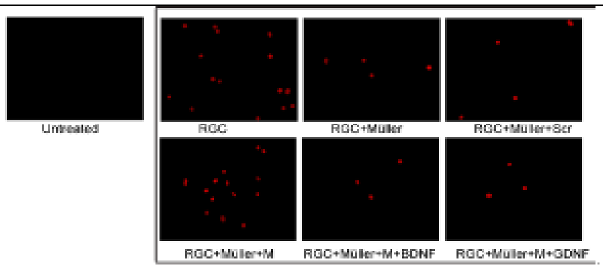

Repeat 2

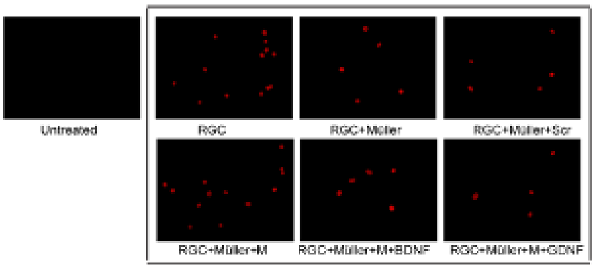

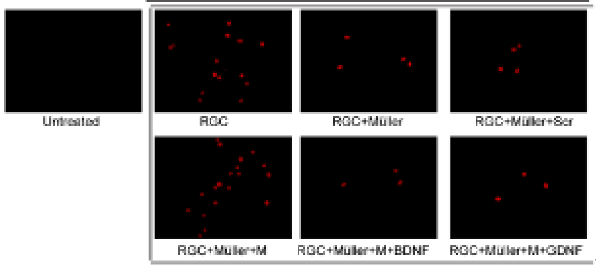

Repeat 3

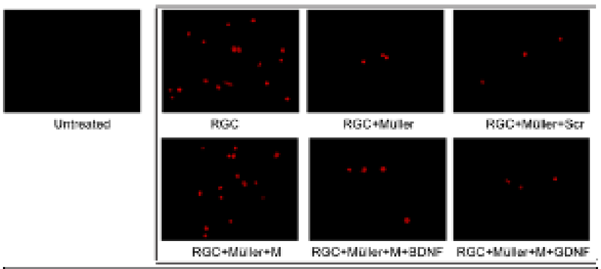

Repeat 4

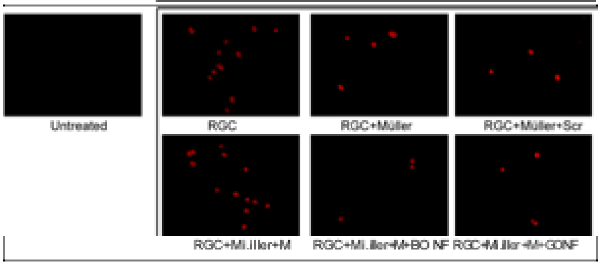

Repeat 5	

For S2: Detection of PKCα expression in ONT

Problem:

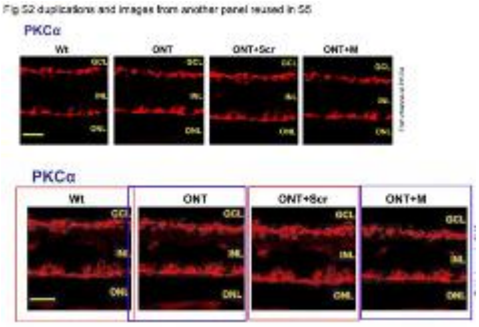

Response:

Four-month old male C57Bl/6J mice were received an intravitreous injection of scrambled (Scr) shRNA or MALAT1 shRNA, or left untreated for 1 week, and then ONT models were established. Two weeks after ONT, retinal slices were immunolabeled for the marker protein, PKCα. This experiment included 4 groups: untreated group (Wt), ONT (traumatic injury), ONT+Scramble shRNA (Scr), and ONT+MALAT1 shRNA (M). The result showed that MALAT1 knockdown did not

affect bipolar cells in ONT retinas. **This result can repeat the published data and is consistent with the previous conclusion** (Red: PKCa Blue: DAPI).

Experimental results:

Repeat 1<br>Repeat 2<br>Repeat 3<br>Repeat 4<br>Repeat 5	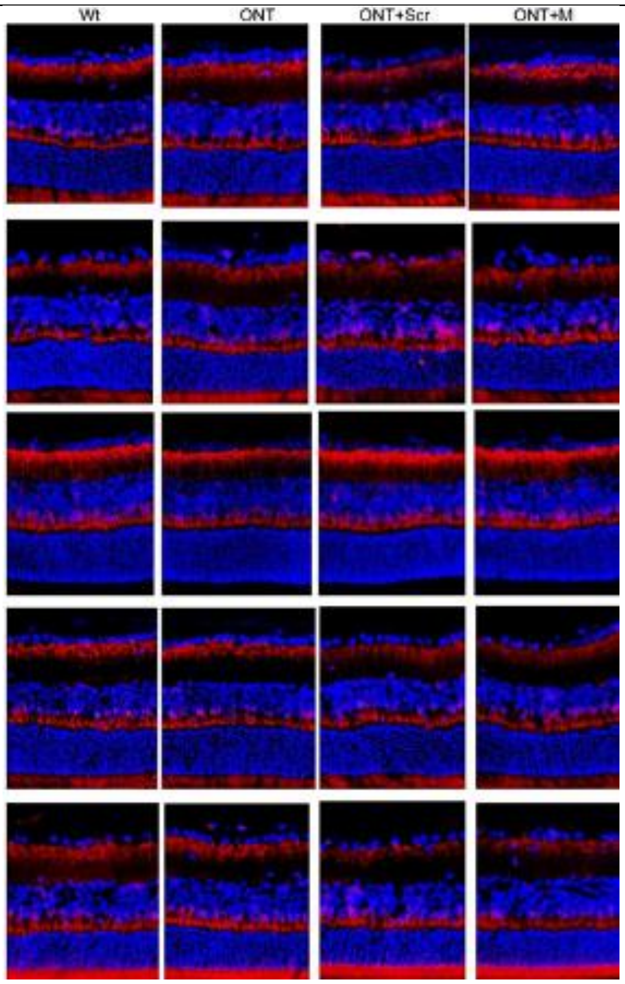

For S2 and S5: Rhodopsin detection

Problem

Fig S2

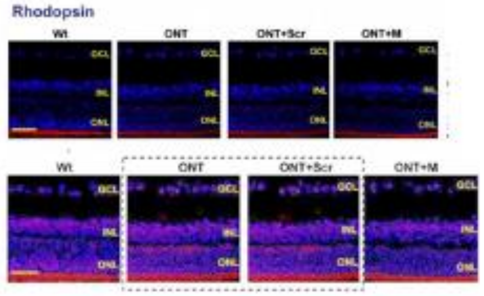

Fig S5

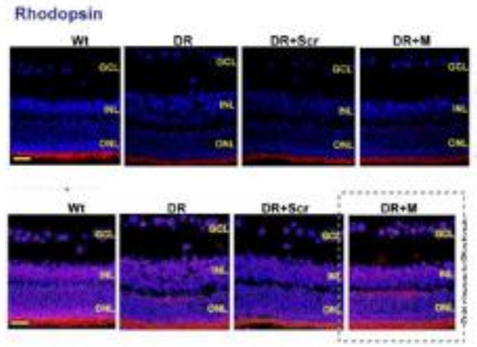

Response:

Four-month old male C57Bl/6J mice were received an intravitreous injection of scrambled (Scr) shRNA or MALAT1 shRNA, or left untreated for 1 week, and then ONT models were established. Two weeks after ONT, retinal slices were immunolabeled for rhodopsin. This experiment included 4 groups: untreated group (Wt), ONT (traumatic injury), ONT+Scramble shRNA (Scr), and ONT+MALAT1 shRNA (M). Rhodopsin immunolabeling revealed that MALAT1 knockdown had no

effect on photoreceptors in ONT retinas. **This result can repeat the published data and is consistent with the previous conclusion.**

Experimental results:

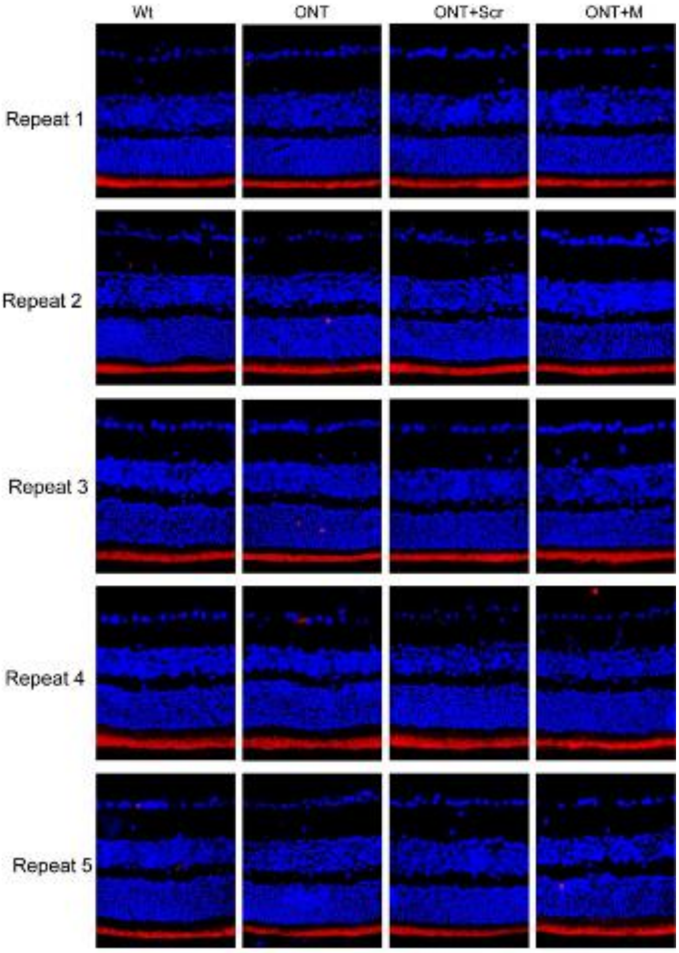

Three-month old SD rats were received an intravitreous injection of scrambled (Scr) shRNA or MALAT1 shRNA, or left untreated for 1 week, and then diabetic models were established. Six months after diabetes induction, retinal slices were immunolabeled for rhodopsin. This experiment included 4 groups: untreated group (Wt), DR (Diabetic group), DR+Scramble shRNA (Scr), and DR+MALAT1 shRNA (M). Rhodopsin immunolabeling revealed that MALAT1 knockdown had no effect on

photoreceptors in diabetic retinas. **The result can repeat the published data and is consistent with the previous conclusion.**

Experimental results:

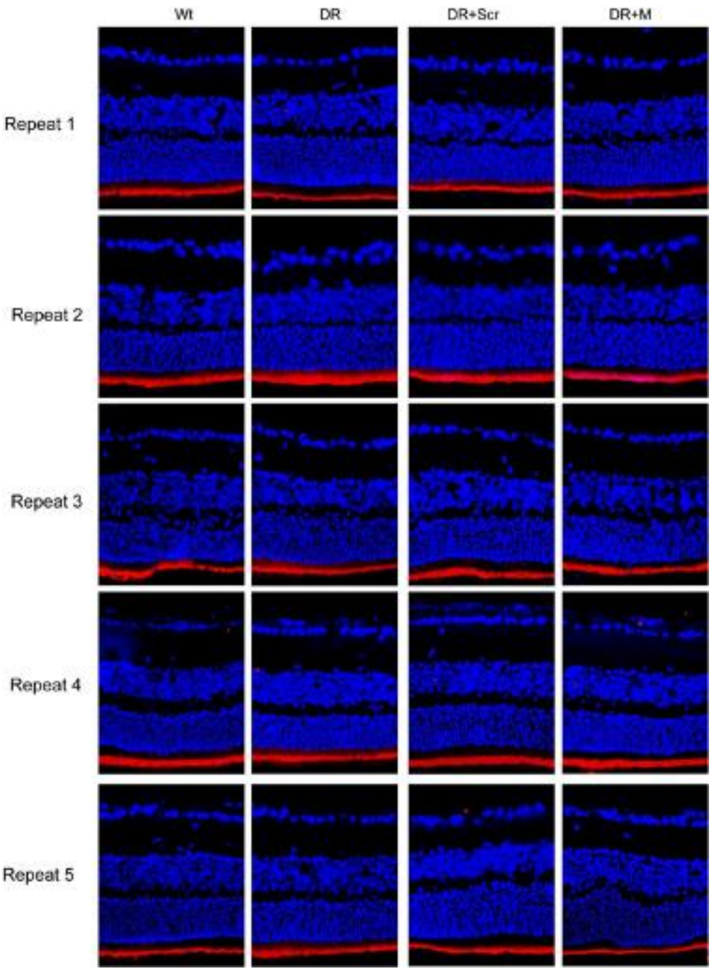

For S3: Detection of GFAP expression in ONT

Problem:

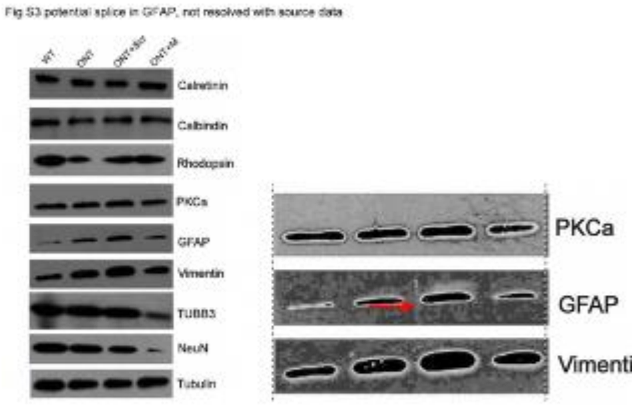

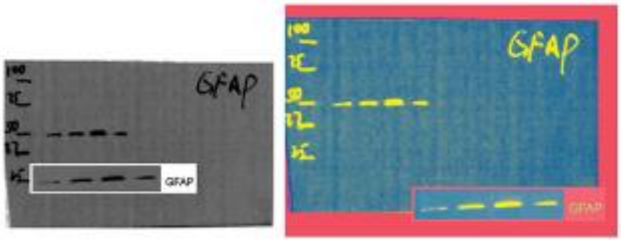

Response:

Four-month old C57Bl/6J mice were received an intravitreous injection of scrambled (Scr) shRNA or MALAT1 shRNA, or left untreated for 1 week, and then ONT models were established. Two weeks after ONT, total proteins were extracted from ONT and wild-type (Wt) retinas. Western blots were performed to detect protein expression. Tubulin was detected as the internal control. This experiment included 4 groups: WT, ONT, ONT+Scr shRNA (ONT+Scr), and ONT+MALAT1 shRNA (ONT+M). Western blots revealed that MALAT1 knockdown reduced GFAP expression levels. The result can repeat the published data and is consistent with the previous conclusion.

Experimental results:

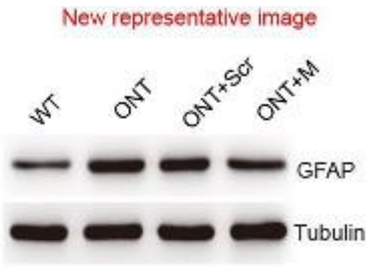

Six repeats (original data)

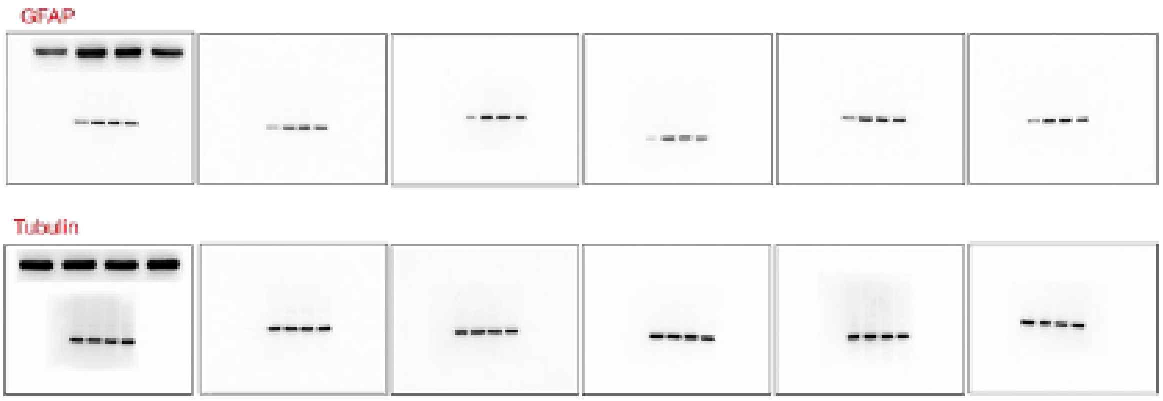

For S5: Detection of PKCα expression in DR

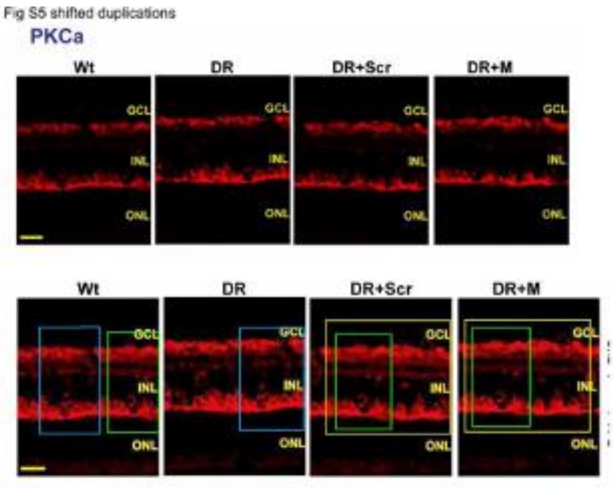

Response:

Three-month old male SD rats were received an intravitreous injection of scrambled (Scr) shRNA or MALAT1 shRNA, or left untreated for 1 week, and then diabetic models were established. Six months after diabetes induction, retinal slices were immunolabeled for the marker protein PKCα. This experiment included 4 groups: untreated group (Wt), DR (diabetic group), DR+Scramble shRNA (Scr), and DR+MALAT1 shRNA (M). The result showed that MALAT1 knockdown did not affect

bipolar cells in diabetic retinas. **The result can repeat the published data and is consistent with previous conclusion (Red: PKCa Blue: DAPI).**

Experimental results:

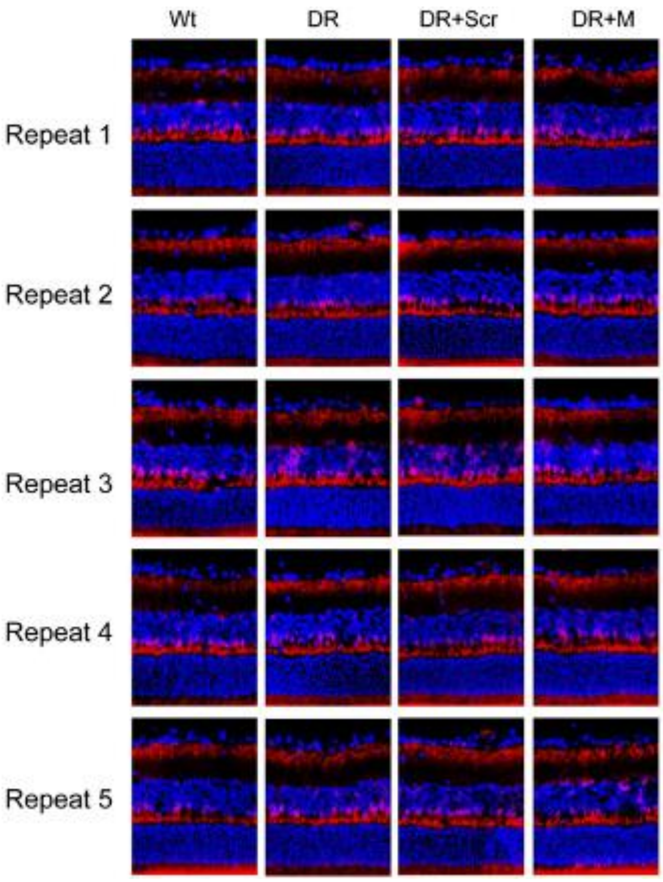

For S8A: Detection of Nestin and Vimentin in ONT

Problem:

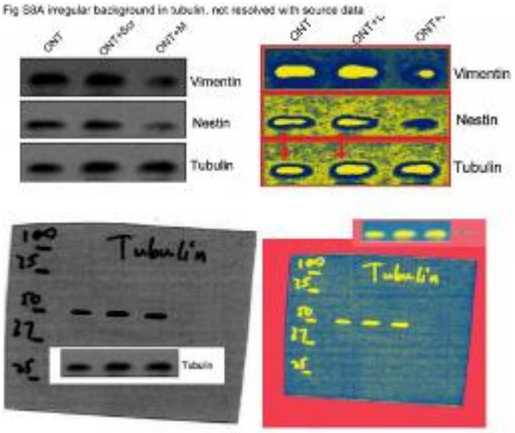

Response:

Four-month old male C57Bl/6J mice were received an intravitreous injection of scrambled (Scr) shRNA or MALAT1 shRNA, or left untreated for 1 week, and then ONT models were

conducted. Two weeks after ONT, the total protein was extracted. Western blots were performed to detect vimentin and nestin expression.

This experiment included 3 groups: ONT, ONT+Scr shRNA (ONT+Scr), and ONT+MALAT1 shRNA (ONT+M). This experiment was re-conducted by two different students and repeated for 3 times. The result showed that MALAT1 knockdown decreased nestin and vimentin expression in the ONT retinas. **The result can repeat the published data and is consistent with the previous conclusion.**

Experimental results:

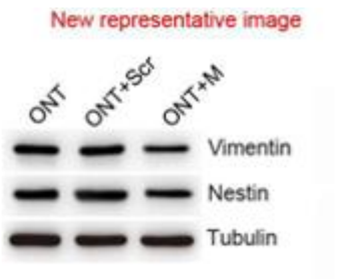

Six repeats (Original data)

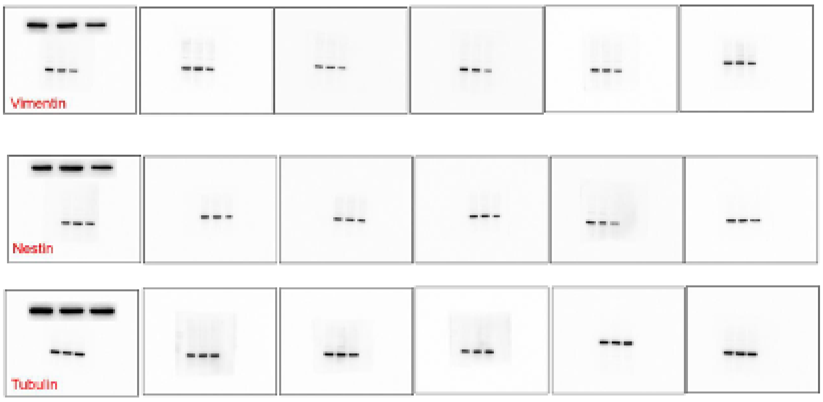

Editor to referee **11th April 2022**

I am contacting you regarding the study by Biao Yan and colleagues (EMM-2016-05725 "Long non-coding RNA MALAT1 regulates retinal neurodegeneration through CREB signaling"), which you reviewed some time ago for EMBO Molecular Medicine. The editorial office has recently become aware of potential image aberrations in this paper, including duplications within figures and between figures 3B,7C, 7D, S2, and S5.

In line with the journal policy, we had notified the research institution for quality control at the institutional level and requested the authors to repeat the experiments under the supervision of the investigative committee. Meanwhile, we have issued an editorial note attached to the

paper to alert readers to these image aberrations and an ongoing effort from the authors to repeat the experiments (https://www.embopress.org/doi/full/10.15252/emmm.202115623).

The authors have now repeated the experiments, provided new figures and analyses. We have conducted a forensic image analysis on the repeat data and did not detect aberrations in these new images. Since the errors occur in figures that are essential to the main conclusion of the paper(specifically Figure 7), I would be grateful if you could take a look at the new figures and analyses and let us know whether you think that they seem acceptable or if, in your opinion, the main conclusions and findings are compromised.

In particular, I would like to point you to several issues we noticed in the new data, and we would appreciate your input on them:

1. In their previous paper (https://www.embopress.org/doi/full/10.15252/emmm.201505725), the authors used Mann-Whitney U-test to calculate the p-value. In their repeat experiments, they performed One-way ANOVA tests. In your view, is the statistical method switch acceptable?

2. In the legend of Figure S2: the authors provided p-values for ONT+scr vs. WT and ONT+M vs. WT, instead of comparing ONT+scr and ONT+M to ONT. Similarly, in Figure S5, they compared DR+ scr or DR+M with WT. Do you think such comparison and analysis are appropriate?

3. In the Rhodopsis experiments in the original Appendix Figures S2 and S5: WT shows a significant difference from the other tested samples. However, WT is not significantly different from the other experiments in the repeat experiments. Is this discrepancy concerning to you, or do you think the authors can clarify this?

I have attached here the original paper, the original appendix file, and the repeat data provided by the authors. Please feel free to let me know if you need additional information.

I am sorry to bother you again with the same manuscript, and we would be very grateful if you could provide us with your expert opinion on this matter.

Referee to Journal **26th April 2022**

I've looked over the new/reproduced data and it seems okay as far as fitting/supporting with their original conclusions. To address each of your questions, here are my thoughts:

1. In their previous paper (https://www.embopress.org/doi/full/10.15252/emmm.201505725), the authors used Mann-Whitney U-test to calculate the p-value. In their repeat experiments, they performed One-way ANOVA tests. In your view, is the statistical method switch acceptable?

They are looking at multiple groups, so Mann-Whitney was not the correct test to be used. However, their previous use of Mann-Whitney suggests that their data does not follow a normal distribution, so the Kruskal-Wallis test would be the appropriate choice instead of ANOVA. I would have thought ANOVA would be fine and a normal distribution could be expected, but perhaps not with the ONT and siRNA injections. I think it might be good for

you to check briefly with a statistician if that's possible, just to see if they should use ANOVA or Kruskal-Wallis. Also, was a multiple comparison's test used? The authors should include that information.

2. In the legend of Figure S2: the authors provided p-values for ONT+scr vs. WT and ONT+M vs. WT, instead of comparing ONT+scr and ONT+M to ONT. Similarly, in Figure S5, they compared DR+ scr or DR+M with WT. Do you think such comparison and analysis are appropriate?

Since the fluorescence intensity is not different between WT or ONT, it won't make a difference. I think showing the change from WT is good, but they should include the p-values for all groups compared/tested, which will include the original comparison. They will have all of that information already if they used ANOVA. Based on the graph values, it shouldn't change any significance shown in their new figure.

3. In the Rhodopsis experiments in the original Appendix Figures S2 and S5: WT shows a significant difference from the other tested samples. However, WT is not significantly different from the other experiments in the repeat experiments. Is this discrepancy concerning to you, or do you think the authors can clarify this?

This one is interesting, and I apologize that I didn't catch it years ago when I reviewed the paper. Based on their results/conclusions in the manuscript, the WT should not have been different. Unless optic nerve transection causes a loss of the photoreceptor cells, which I'm not aware of from my experience in the field. I believe the new data provided is more accurate, but the authors should clarify the discrepancy in those results and why they initially had a significant decrease from WT.

Additional Comments:

ONT is mislabeled in the graph for Supplementary figure 2 with the PKCa staining.

For figure 7D, can they make sure to include how they counted the abnormal axons? Was it just by eye from an investigator for the 3 images they took per group? Or was software used to detect the abnormalities? Did they add up the abnormalities from all three images or average them? I'm guessing add, since they have a max of approximately 40 for the new data and almost 90 for the published figure (but used 10 images instead of three for the analysis). They should make sure to state how they counted and whether the investigator was blinded or aware of the groupings.

Overall, I don't see any modifications that would alter their original conclusions. Please let me know if you want me to look closer at any of the other figures/data in the manuscript, I focused on the figures where they provided new data.

Editor to statistics expert — 29th April 2022

I hope this note finds you well. If you have the time, I would appreciate your advice on a statistical issue of a manuscript published in EMBO Molecular Medicine in 2016 (https://www.embopress.org/doi/full/10.15252/emmm.201505725).

We recently became aware of potential image aberrations in this paper, so we asked the authors to repeat some of the experiments. The authors now repeated the experiments and provided new figures and analyses. However, in their previous article, the authors used Mann-Whitney U-test, and in their repeat experiments, they performed One-way ANOVA

tests. We asked one of the reviewers who reviewed the original paper to comment on this, and here is their response:

"They are looking at multiple groups, so Mann-Whitney was not the correct test to be used. However, their previous use of Mann-Whitney suggests that their data does not follow a normal distribution, so the Kruskal-Wallis test would be the appropriate choice instead of ANOVA. I would have thought ANOVA would be fine and a normal distribution could be expected, but perhaps not with the ONT and siRNA injections. I think it might be good for you to check briefly with a statistician if that's possible, just to see if they should use ANOVA or Kruskal-Wallis. Also, was a multiple comparison's test used? The authors should include that information."

In light of this comment, I would appreciate your advice on

- whether the new statistic test (ANOVA) is appropriate for this type of data? If not, which test is most suitable?

- Were the statistics (Mann-Whitney U-test) used in the original paper and the data interpretation completely flawed? Or, although the previous test was not optimal, the data interpretation is still acceptable?

I have attached here the new data provided by the authors. Please let me know if you need additional information.

Thank you very much in advance. I would be very grateful if you could provide us with your expert opinion on this matter.

Statistics expert to Journal **3rd May 2022**

One-way ANOVA between two groups is the t-test. Looking at the plots, this looks fine. Also, it should not matter — qualitatively similar results should also come out with the Mann-Whitney U-test. But for several theoretical and conceptual reasons, the t-test (or ANOVA test) is actually preferable.

I think the statement "They are looking at multiple groups, so Mann-Whitney was not the correct test to be used." is not pertinent, since even although there are >2 groups, the comparisons are always pairwise.

Perhaps, to make sure your readers do not get the same (unfounded) doubts as your reviewer, the authors could be asked to provide the p-values for that, too. The basic results should be consistent.

Journal's response to authors **7th June 2022**

Thank you for sending us your repeat data and analysis. We have now gone through your data and response in detail. As you will see below, we have also invited one of the referees who had evaluated the original manuscript to review the repeat data. In particular, we asked them specifically to comment on several issues that we noticed.

I have included below our questions and the referee's response. Considering these comments, we would ask you to address the following issues:

- Point #2: please include the p-values for all groups compared/tested.
- Point #3: please clarify the discrepancy in those results.
- Address the additional comments from the referee about Figure 7.
- Please note that for point #1, we also consulted with a statistician who confirmed that the current one-way ANOVA test is appropriate.

Please send us a revised version of the repeat data and a point-by-point response to the referee's comments. Feel free to let me know if you have any questions.

Author's response **20th June 2022**

Thank you for sending us your repeat data and analysis. We have now gone through your data and response in detail. As you will see below, we have also invited one of the referees who had evaluated the original manuscript to review the repeat data. In particular, we asked them specifically to comment on several issues that we noticed.

I have included below our questions and the referee's response. Considering these comments, we would ask you to address the following issues:

-Point #2: please include the p-values for all groups compared/tested.

Response:

We all included the p-values for all groups compared/tested for figure S2 and figure S5.

Fig. S2: MALAT1 knockdown did not affect photoreceptors in ONT retinas

Four-month old male C57Bl/6J mice were received an intravitreous injection of scrambled (Scr) shRNA or MALAT1 shRNA, or left untreated for 1 week. Then, ONT models were established. Two weeks after ONT, retinal slices were immunolabeled for the marker protein, rhodopsin. n = 5 animals per group independent experiments; One-way ANOVA; **P* = 0.934 (ONT versus WT), **P* = 0.584 (ONT+Scr versus WT), **P* = 0.387 (ONT+M versus WT); **P* = 0.562 (ONT+Scr versus ONT), **P* = 0.380 (ONT+M versus ONT), **P* = 0.897 (ONT+M versus ONT+Scr); Red: rhodopsin; Blue: DAPI.

Fig. S5: MALAT1 knockdown did not affect photoreceptors in diabetic retinas

Three-month old male SD rats were received an intravitreous injection of scrambled (Scr) shRNA or MALAT1 shRNA, or left untreated for 1 week. Then, the diabetic models were established. Six months after diabetes induction, retinal slices were immunolabeled for the marker protein, rhodopsin. n = 5 animals per group; One-way ANOVA; *P = 0.506 (DR versus Wt), *P = 0.663 (DR+Scr versus Wt), *P = 0.783 (DR+M versus Wt); *P = 0.312 (DR+Scr versus DR), *P = 0.718 (DR+M versus DR), *P = 0.524 (DR+M versus DR+Scr); Red: rhodopsin; Blue: DAPI.

-Point #3: please clarify the discrepancy in those results.

Response:

We first explained why Rhodopsin expression in WT group was altered in the repeated experiment.

Rhodopsin is a G-protein coupled receptor found in the rod cells of the retina. As a biomarker associated with retinal thinning and degeneration, it shows the potential in the early detection and monitoring of several neurodegenerative diseases (Lenahan et al., Front Neurosci 2020; Smith, Annu Rev Biophys 2010). By contrast, other researchers have reported that Rhodopsin mRNA, which was expressed constitutively at a high level in the retina, is known to be unaffected in the early period following stress and can function as a useful endogenous control transcript under a variety of circumstances (Glyn et al., Molecular Vision 2005; Standfuss et al., Nature 2011). These evidences indicate that Rhodopsin is expressed constitutively at a high level. Its expression could be altered or unaltered, which is dependent on the severity of the external injuries.

In diabetic model, as for the expression of Rhodopsin, different researcher have shown different results. Streptozotocin-induced diabetic rats showed decreased a- and b-wave amplitudes of scotopic and photopic electroretinography responses 4 months after diabetes induction compared to nondiabetic controls. Western blot analysis did not reveal the expression change of opsin but reveal a sight expression reduction in rhodopsin in diabetic retinas. Rhodopsin regeneration and homeostasis are critical for retinal function and health. In fact, the visual chromophore 11-cis-retinal binds to opsin to form rhodopsin in the dark, which locks the opsin in an inactive state. Unbound free opsin is known to be constitutively active, which can exhaust photoreceptor cells and promote retinal degeneration (Park Adv Pharmacol 2014; Malechka et al., Am J Pathol 2017). However, in another group, they found that Rhodopsin levels in WT and Lrat+/- diabetics were similar to corresponding values of 11-cis-retinal as determined by retinoid analyses when compared to WT nondiabetics (ARVO

Annual Meeting, 2022). Hick's lab also reported that there was no significant changes in rhodopsin levels between control and diabetic animals (Yamamoto et al., Br J Ophthalmol 1996; Hicks et al., Invest Ophthalmol Vis Sci 2011). Retinal vein occlusion (RVO) results from the compression of the retinal vein as a result of atherosclerosis or increased blood viscosity (Khayat et al., Surv Ophthalmol 2018). RVO has some similar pathological features as diabetic retinopathy such as ischemic insult. In one study, RVO was induced in pigs, which led to a reduction in proteins involved in vision, such as rhodopsin (Lenahan et al., Front Neurosci 2020). These studies indicate that Rhodopsin expression may be down-regulated or unchanged in diabetic condition although these researchers used the similar animals or detected at the similar time points.

Previous studies about Rhodopsin expression in ONT model. Optic nerve transection (ONT) is a valuable model for investigation into pathways that contribute to RGC death. Death of RGCs does not commence until approximately 5 days following ONT, then, between day 5 and day 14, there is massive loss of RGCs and by day 14 only approximately 15% of the RGCs remain in the retina. Generally, the primary injury sites of ONT model are RGCs (Nickells. Brain Res Bull 2004; Kermer et al., Brain Res Brain Res Protoc 2001; Berkelaar et al., J Neurosci 1994).

Some studies have reported the expression pattern of rhodopsin in ONT model. One study has reported that ONT treatment did not alter the expression of rhodopsin (Chidlow et al., Mol Vis). By contrast, various insults (mechanical injury, bright light, and ischemia) have been shown to protect the retina against subsequent light-induced photoreceptor degeneration. For example, Rod opsin mRNA levels and rhodopsin protein levels were also significantly up-regulated in the axotomized and NMDA-treated eyes compared with the sham-treated fellow eyes after the injury (Casson et al., IOVS 2004). In addition, other works has shown that rhodopsin, opsin, or recoverin, were transiently down-regulated as soon as 12 h after ONT, when gene transcription is severely halted and mRNA levels diminish to approximately one half of their normal values. Interestingly, the basal values of these mRNAs recover slowly within the next weeks (Lindqvist et al., Brain Res Brain Res Protoc 2002; Agudo et al., Mol Vis. 2008). These evidences suggest that the expression of Rhodopsin is also highly variable in ONT model.

Taken together, both DR model and ONT model, the expression of Rhodopsin is highly variable. Its expression is affected by different species, different batches, different operators, different time points, or difficult culture conditions. Both diabetes and ONT treatment may not directly affect the expression of Rhodopsin. Thus, different researchers have detected different expression pattern of Rhodopsin.

In the repeated experiment, the experiment was conducted by a different student. The previous student has been graduated and leaved. We also have moved our lab and the animal culture condition was also changed. Thus, these external factors may affect the expression of Rhodopsin in WT group.

As for Rhodopsin experiments in Figures S2 and S5, the purpose of these experiments was to investigate whether MALAT1 knockdown affected the photoreceptors in diabetic retinas or ONT mice. The expression of Rhodopsin between ONT+M group and ONT group or between ONT+M group and ONT+Scr group did not show significant difference. The expression of Rhodopsin between DR+M group and DR group or between DR+M group and DR+Scr group did not show significant difference. Based on the above-mentioned results, we could conclude that the final conclusion was not associated with WT group. And, the current result did not alter the total conclusion of this manuscript.

-Address the additional comments from the referee about Figure 7.

Response:

Three ultra-thin cross sections per nerve were observed and added together to count the number of damaged axons. Counting of damaged axons was performed by three different investigators blinded to group identity and injury status. An average counting number of the three investigators was used for statistical analysis.

-Please note that for point #1, we also consulted with a statistician who confirmed that the current one-way ANOVA test is appropriate.

Response:

According to the suggestion of the statistician, the t-test (or ANOVA test) is actually preferable, we used the One-way ANOVA followed by Dunnet's multiple comparison test for statistical analysis.

Additional comments from the reviewer

"ONT is mislabeled in the graph for Supplementary figure 2 with the PKCa staining.

Response:

We have corrected it. “NOT” has been changed to “ONT”

For figure 7D, can they make sure to include how they counted the abnormal axons? Was it just by eye from an investigator for the 3 images they took per group? Or was software used to detect the abnormalities? Did they add up the abnormalities from all three images or average them? I'm guessing add, since they have a max of approximately 40 for the new data and almost 90 for the published figure (but used 10 images instead of three for the analysis). They should make sure to state how they counted and whether the investigator was blinded or aware of the groupings.

Response:

Three ultra-thin cross sections per nerve were observed and added together to count the number of damaged axons. Counting of damaged axons was performed by 3 different investigators blinded to group identity and injury status. An average counting number of the 3 investigators was used for statistical analysis.

Journal's response 15th July 2022

Thank you for sending us the most recent point-by-point. We are satisfied with the repeat data and plan to move forward to correct your paper.

We would selectively retract Figures 3B, 7C-D, Appendix Figures S2 and S5, and publish the repeat data in the retraction notice. We cannot replace the data in the original manuscript because the repeat experiments were not part of the original experimentation.

We've drafted a partial retraction note (attached) and have included space for you to make an author statement. We would ask you to return the document with your modifications by 29th of July. After we have agreed on a final draft, we will send the notice to all authors and request that everyone signals whether they agree or disagree with the notice and the partial retraction.

In addition, we would require the figure legends included in the word document, and production-quality figures for the repeat data.

Thank you for your collaboration to come to a resolution on this case.

Author's Response **17th July 2022**

Dear editor:
Thanks for giving us the chance for correction.

We have provided the relevant files in the attachment, including
(1) a partial retraction note
(2) Figure legend
(3) production-quality figures for the repeat data for Figures 3B, 7C-D, Appendix Figures S2 and S5
Thanks!
Biao
